# TGF-β3 Inhibits Antibody Production by Human B Cells

**DOI:** 10.1371/journal.pone.0169646

**Published:** 2017-01-04

**Authors:** Yumi Tsuchida, Shuji Sumitomo, Kazuyoshi Ishigaki, Akari Suzuki, Yuta Kochi, Haruka Tsuchiya, Mineto Ota, Toshihiko Komai, Mariko Inoue, Kaoru Morita, Tomohisa Okamura, Kazuhiko Yamamoto, Keishi Fujio

**Affiliations:** 1 Department of Allergy and Rheumatology, Graduate School of Medicine, The University of Tokyo, Tokyo, Japan; 2 Laboratory for Autoimmune Diseases, Center for Integrative Medical Sciences, RIKEN, Yokohama, Japan; 3 Max Planck-The University of Tokyo Center for Integrative Inflammology, The University of Tokyo, Tokyo, Japan; Seoul National University College of Pharmacy, REPUBLIC OF KOREA

## Abstract

TGF-β is a pleotropic cytokine involved in various biological processes. Of the three isoforms of TGF-β, TGF-β1 has long been recognized as an important inhibitory cytokine in the immune system and has been reported to inhibit B cell function in both mice and humans. Recently, it has been suggested that TGF-β3 may play an important role in the regulation of immune system in mice. Murine CD4^+^CD25^-^LAG3^+^ regulatory T cells suppress B cell function through the production of TGF-β3, and it has been reported that TGF-β3 is therapeutic in a mouse model of systemic lupus erythematosus. The effect of TGF-β3 on human B cells has not been reported, and we herein examined the effect of TGF-β3 on human B cells. TGF-β3 suppressed B cell survival, proliferation, differentiation into plasmablasts, and antibody secretion. Although the suppression of human B cells by TGF-β1 has long been recognized, the precise mechanism for the suppression of B cell function by TGF-β1 remains elusive; therefore, we examined the effect of TGF-β1 and β3 on pathways important in B cell activation and differentiation. TGF-β1 and TGF-β3 inhibited some of the key molecules of the cell cycle, as well as transcription factors important in B cell differentiation into antibody secreting cells such as IRF4, Blimp-1, and XBP1. TGF-β1 and β3 also inhibited B cell receptor signaling. Our results suggest that TGF-β3 modifying therapy might be therapeutic in autoimmune diseases with B cell dysregulation in humans.

## Introduction

Transforming growth factor-beta (TGF-β) is a pleotropic cytokine involved in various biological processes. There are three isoforms of TGF-β in mammals[1]. Each isoform is thought to have different biological roles *in vivo* as the expression of the three isoforms differ in their pattern of expression and knock out mice of different isoforms exhibit different phenotypes[2, 3]. TGF-β1 knock out mice develop autoinflammatory disease characterized by inflammation in various organs and production of autoantibodies[4, 5]. TGF-β2 knockout mice exhibit various congenital abnormalities involving the cardiovascular, pulmonary, skeletal, and urogenital systems[3], and TGF-β3 knockout mice exhibit cleft palate and delayed lung development[3]. In certain contexts, different isoforms exhibit opposing effects. For example, TGF-β1 promotes fibrosis during wound healing, but TGF-β3 has anti-fibrotic effects[6–8].

Of the three isoforms of TGF-β, TGF-β1 had mainly received attention in immunology until recently and is generally known as an inhibitory cytokine, although it exhibits immunostimulatory functions in certain conditions[9]. TGF-β1 inhibits proliferation of T cells, as well as T cell differentiation into Th1 cells and Th2 cells[9]. TGF-β1 also inhibits excessive immune response by promoting induction and maintenance of Foxp3^+^ regulatory T cells (Treg cells)[9], and TGF-β1 contributes to the immunosuppressive function of Foxp3^+^ Treg cells[9]. However, TGF-β1, when present with inflammatory cytokines, may promote inflammation by promoting the differentiation of Th17 cells[9].

TGF-β1 has profound effects on B cells as well and has been reported to inhibit proliferation and antibody production of B cells in both mice and humans[10–13]. However, in certain contexts, TGF-β1 induces proliferation of B cells and IgA production[12, 14–16]. *In vivo*, TGF-β1 is expressed on the surface of Foxp3^+^ Treg cells and is involved in the inhibition of B cells by Foxp3^+^ Treg cells[17]. However, studies of the mechanism of B cell inhibition by TGF-β1 on human B cells have mainly been conducted in the context of oncology using cell lines with a focus on oncogenes and apoptosis related genes[18–22]. Thus, our knowledge of the effect of TGF-β1 on signal pathways in primary human B cells, especially on pathways important in antibody production, is limited.

Until recently, the role of TGF-β3 in vivo has mainly been described in development with little focus on the immune system[1], but there is emerging evidence from studies in mice that TGF-β3 is also important in the regulation of the immune system. For instance, Th17 cells induced in the presence of TGF-β3 exhibit different characteristics from Th17 cells induced in the presence of TGF-β1[23]. In addition, TGF-β3 produced by resting B cells induce proliferation of Foxp3^+^ Treg cells[24]. TGF-β3 is also produced by CD4^+^CD25^-^LAG3^+^ Treg cells, IL-10 producing Treg cells characterized by the expression of early growth response protein-2 and lymphocyte activation gene 3 (LAG3) protein[25, 26]. LAG3^+^ Treg cells suppress B cells through the production of TGF-β3[25]. The administration of TGF-β3 expressing vector to MRL/*lpr* mouse, a mouse model of systemic lupus erythematosus (SLE), ameliorated the progression of nephritis. Thus, TGF-β3 modifying therapy might be therapeutic in autoimmune diseases with B cell dysregulation[25].

We herein examined the effect of TGF-β3 on human B cells, which has not yet been reported. Like TGF-β1, TGF-β3 suppressed B cell survival, proliferation, differentiation into antibody-secreting cells (ASCs), and antibody production. To elucidate the mechanism for inhibition of human primary B cells by TGF-β1 and β3, we performed transcriptome analysis using RNA-Sequencing (RNA-Seq) and subsequent pathway analysis, followed by further analysis of some of the key molecules.

## Materials and Methods

### Cell Isolation and Culture

Peripheral blood mononuclear cells (PBMCs) were separated from heparinized whole blood by density gradient centrifugation using Ficoll-Paque PLUS (GE Healthcare). B cells were purified using Human B Cell Isolation Kit II (Miltenyi Biotec), and naïve B cells were isolated using Human Naïve B Cell Isolation Kit (Miltenyi Biotec). The ethics committee of the University of Tokyo Hospital approved this study (No. 10154 and G3582). All subjects provided written informed consent, and the study was conducted in accordance with relevant guidelines.

Unless otherwise indicated, cells were cultured in RPMI 1640 (Invitrogen) supplemented with 10% FCS (Equitech Bio), 100 μg/ml L-glutamine, 100 U/ml penicillin, 100 μg/ml streptomycin (Invitrogen), and 50 μM 2-ME (Sigma). In some experiments, cells were cultured in X-VIVO15 (Lonza) to exclude the effect of TGF-β in FCS.

TGF-β1 and β3 (R&D) were used at 1 ng/ml unless otherwise indicated. IL-21 (PeproTech), IL-4 (R&D), soluble CD40L (PeproTech), and CpG-ODN2006 (Enzo Life Sciences) were used at 50 ng/ml, 100U/ml, 2 μg/ml, and 6 μg/ml respectively, and BCR stimulation was induced using goat anti-human IgA + IgG + IgM (H+L) (Jackson ImmunoResearch) at 2.5 μg/ml.

### Antibody Production

B cells and PBMCs were cultured at 3x10^5^/well in 96 well plates. ELISA was performed using Human IgG ELISA Quantitation Set, Human IgA ELISA Quantitation Set, and Human IgM ELISA Quantitation Set (Bethyl Laboratories).

### Assessment of Cell Proliferation

Cells were suspended in 2% FCS-containing medium at 1 x 10^7^/ml, and CFSE (Dojindo) was added to achieve a final concentration of 2 μM. After incubating for 5 minutes at room temperature, cells were washed with 100% FCS and cultured in 10% FCS-containing medium.

### Flow Cytometry

Human Fc Receptor Binding Inhibitor Purified (eBioscience) was used to block unspecific Ab binding, and the cells were stained with the following monoclonal Abs: APCCy7-CD19 (HIB19, BioLegend), APC-CD38 (HIT2, Biolegend), and PECy7-CD38 (HIT2, Biolegend). For the quantification of light chains on the cell surface, mouse anti-human Ig κ light chain (G20-193, BD), mouse anti-human Ig λ light chain (JDC-12, BD), and APC rat anti-mouse IgG1 (A85-1, BD) were used. 7-Amino-Actinomycin D (Biologend) was used to assess cell death. For intracellular staining of Blimp-1, Blimp-1 (N-20) AF488 (Santa Cruz) and Foxp3/Transcription Factor Staining Buffer Set (eBioscience) were used. For intracellular staining of phosphorylated STAT3, cells were fixed with 2% paraformaldehyde, permeabilized with 96% methanol, and stained with PE Mouse Anti-Stat3 (pY705, BD). Flow cytometry was performed using MoFlo XDP (Beckman Coulter), and data were analyzed using FlowJo 7.6.5 (Tree Star).

### RNA-Seq

RNA was extracted using RNeasy Micro Kit (Qiagen), and libraries were prepared using TruSeq Stranded mRNA LT Kit (Illumina). Paired-end sequencing was performed using HiSeq 2500 (Illumina). Cutadapt[27] and FASTX-Toolkit (http://hannonlab.cshl.edu/fastx-toolkit) were used to remove adaptor sequences and ends with phred quality scores less than 20. UCSC hg19 reference sequence (http://genome.ucsc.edu/) was used as the reference genome, and STAR[28] was used for mapping. Read count was obtained for each gene using HTSeq[29]. Read count after quality control was 4.9x10^6^ ~ 9.7x10^6^. Differential gene analysis was performed using edgeR 3.12.0[30]. Pathway analysis was performed by uploading genes with false discovery rate less than 0.05 by the Benjamini-Hochberg method and their logFCs into IPA software (Qiagen)[31]. R version 3.2.3 was used for RNA-Seq analysis.

### Quantitative PCR

RNeasy Micro Kit (Qiagen) was used to extract RNA, and cDNA was synthesized using Random Primers (Invitrogen) and Superscript III (Invitrogen). Quantitative PCR was performed using QuantiTect SYBR Green PCR Kit (Qiagen) and CFX Connect Realtime PCR (BioRad). Primers used were as follows: *GAPDH* (Forward: GAAGGTGAAGGTCGGAGTC, Reverse: GAAGATGGTGATGGGATTTC), *IRF4* (Forward: ACCTGCAAGCTCTTTGACAC, Reverse: AAAGCATAGAGTCACCTGGAATC), *PRDM1* (Forward: GTGTCAGAACGGGATGAACA, Reverse: GCTCGGTTGCTTTAGACTGC), *XBP1* (Forward: CCGCAGCACTCAGACTACG, Reverse: TGCCCAACAGGATATCAGACT), *BCL6* (Forward: CTGGCTTTTGTGACGGAAAT, Reverse: AACCTGAAAACCCACACTCG), *PAX5* (Forward: ATCATCCGGACAAAAGTACAGC, Reverse: GTGCTCACCGAGGACACC), *FCRL4* (Forward: GTGAGGGGTAACATCCACAAGC, Reverse: CTTCAGCCACGGAGCAGAC), and *AICDA* (Forward: GACTTTGGTTATCTTCGCAATAAGA, Reverse: AGGTCCCAGTCCGAGATGTA). For the quantification of mature Ig transcripts framework 3 (FR3) forward primer (GACACGGCTGTGTATTACTGTGCG) was used in combination with the following reverse primers: V_H_DJ_H_-C_H_γ_1_ (GTTTTGTCACAAGATTTGGGCTC), V_H_DJ_H_-C_H_γ_2_ (GTGGGCACTCGACACAACATTTGCG), V_H_DJ_H_-C_H_γ_3_ (TTGTGTCACCAAGTGGGGTTTTGAGC), V_H_DJ_H_-C_H_α_1_ (GGGTGGCGGTTAGCGGGGTCTTGG), and V_H_DJ_H_-C_H_α_2_ (TGTTGGCGGTTAGTGGGGTCTTGCA)[32]. Expression relative to *GAPDH* was calculated using the comparative Ct method.

### Western Blotting

Cells were lysed using lysis buffer (50mM Tris HCl pH 7.5, 150mM NaCl, 1% Triton X-100, 1mM EDTA) and denatured with Laemmli Sample Buffer (Biorad) at 95°C for 5 minutes. After SDS-PAGE, proteins were transferred to Immobilon-P Transfer Membrane (Millipore). After blocking with 5% BSA or 5% skim milk, blots were incubated overnight with following primary Abs: phospho-Smad1/5, phospho-Smad2, phospho-Smad3, phospho-Syk, total Syk, phospho-NF-κB p65, total NF-κB (all from Cell Signaling), or Actin (I-19) (SantaCruz). The blots were then incubated with HRP-Goat Anti-Rabbit IgG (H+L) (Zymed or Invitrogen), and bands were detected using ECL Select Western Blotting Detetion Reagent (GE Healthcare). Stripping was performed with Restore PLUS Western Blot Stripping Buffer (Thermo Scientific) or buffer prepared in-house (50 mM 2-ME, 2% SDS, 100 mM Tris-HCl).

### Statistical Analysis

Data are presented as average ± SD. With the exception of RNA-Seq data, data were analyzed using GraphPad Prism 5 (GraphPad Software). For comparison of multiple groups, one way ANOVA and Dunnett test were used. p-values less than 0.05 were considered significant. *, **, ***, and **** indicate p<0.05, p<0.01, p<0.001, and p<0.0001 respectively.

## Results

### TGF-β3 Inhibits Antibody Production by Human B Cells

**Fig 1:**
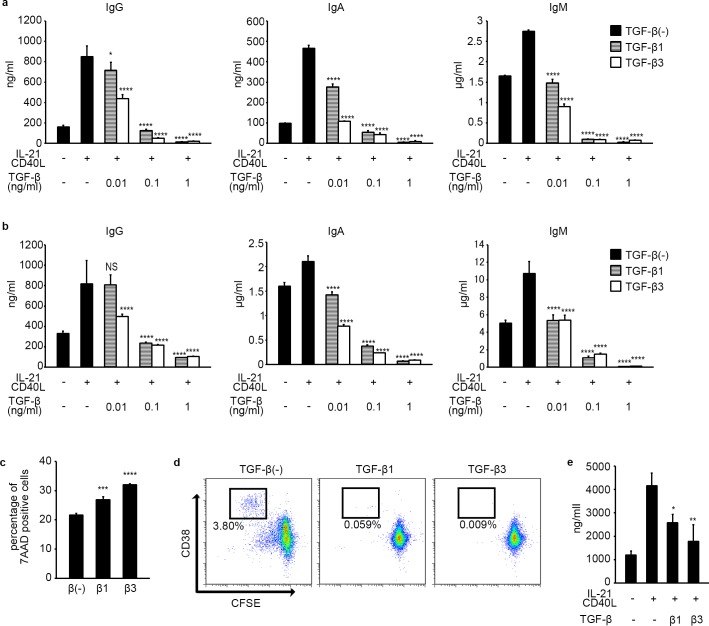
TGF-β3 inhibits B cell antibody production. (a, b) B cells were cultured under IL-21 and CD40L stimulation for 11 days in serum containing medium (a) or serum free medium (b), and antibody production was assessed by ELISA. Among the IL-21 and CD40L stimulated samples, each of the TGF-β treated samples was compared with TGF-β untreated sample using one way ANOVA, Dunnett test (n = 3). Results are representative of two similar experiments. (c) B cells were cultured as in (a) and the percentage of 7AAD positive cells among CD19^+^ cells were assessed by flow cytometry. TGF-β treated samples were compared with TGF-β untreated sample using one way ANOVA, Dunnett test (n = 3). Results are representative of two similar experiments. (d) B cells were cultured under IL-21 and CD40L stimulation for 5 days and cell proliferation and the percentage of CD38^high^ plasmablasts among CD19^+^7AAD^-^ cells were assessed by flow cytometry. Results are representative of two similar experiments. (e) PBMCs were stimulated with IL-21 and sCD40L for 12 days, and IgG production was assessed by ELISA. Among the IL-21 and CD40L stimulated samples, each of the TGF-β treated samples was compared with TGF-β untreated sample using one way ANOVA, Dunnett test (n = 3). Results are representative of three independent experiments.

**Fig 2:**
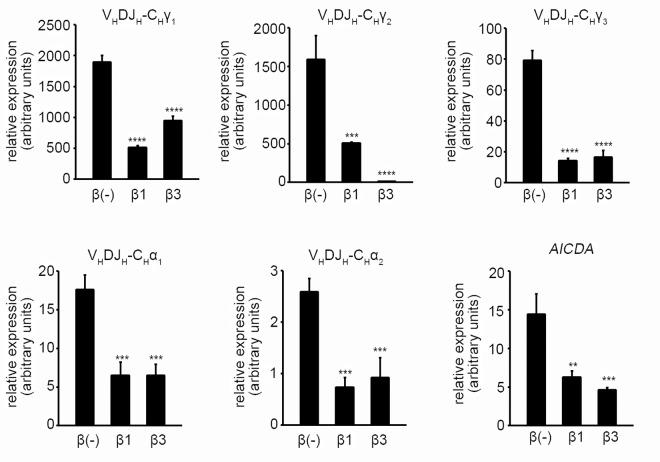
TGF-β1 and β3 inhibit the expression of mature IgG and IgA transcripts and *AICDA*. Naïve B cells were cultured under IL-21, IL-4, and sCD40L stimulation for 4 days, and the expression of mature IgG1, IgG2, IgG3, IgA1, and IgA2 transcripts, as well as *AICDA*, were assessed by quantitative PCR. Expression relative to *GAPDH* is shown. TGF-β treated cells were compared with TGF-β untreated cells using one way ANOVA, Dunnett test (n = 3). Results are representative of two similar experiments.

TGF-β3 inhibited IgG, IgA, and IgM production by human B cells under IL-21 and CD40L stimulation as strongly as TGF-β1 (Fig 1A). Although the majority is in the latent form and not biologically active, FCS has been reported to contain 1–2 ng/ml of TGF-β1[33], and to exclude the effect of TGF-β1 in the FCS, the experiment was conducted in serum free medium with similar results (Fig 1B). TGF-β1 and TGF-β3 induced cell death in human B cells, suggesting that the decrease in antibody production may partly be due to decreased survival of B cells (Fig 1C). In addition, B cell proliferation and differentiation into CD38^high^ plasmablasts were inhibited by TGF-β1 and TGF-β3; therefore, in addition to decreased cell survival, the decrease in antibody production by TGF-β1 and β3 may be due to decreased cell proliferation and differentiation into ASCs (Fig 1D). To assess the effect of TGF-β1 and β3 in a more physiological condition with help from T cells, B cells were cultured along with autologous PBMCs. TGF-β1 and β3 also inhibited IgG production under this condition (Fig 1E). The expression of mature IgG1, IgG2, IgG3, IgA1, and IgA2 transcripts, as well as *AICDA*, were downregulated by TGF-β1 and β3, suggesting that TGF-β1 and β3 suppress class switching and affinity maturation (Fig 2).

**Fig 3:**
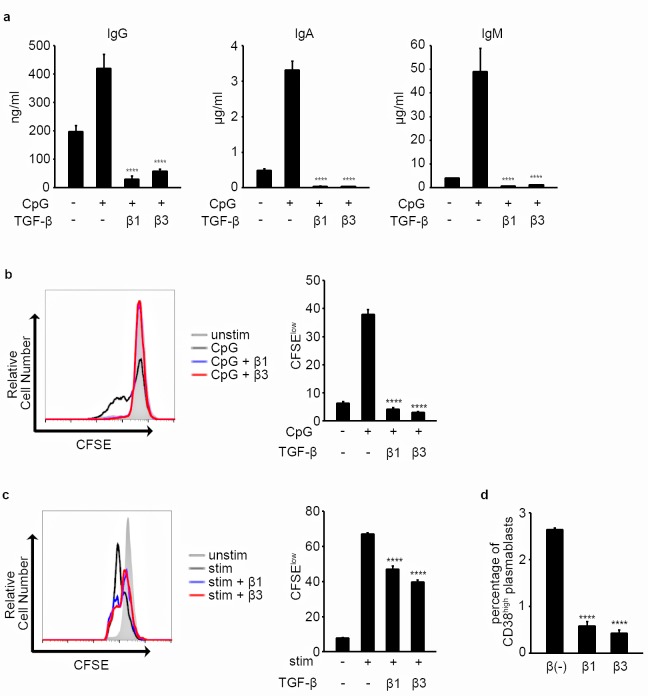
TGF-β1 and β3 inhibit B cell function under various stimulatory conditions. (a) B cells were stimulated with CpG-ODN2006 for 8 days, and antibody production was assessed by ELISA. Among the CpG-ODN2006 stimulated samples, each of the TGF-β treated samples was compared with TGF-β untreated sample using one way ANOVA, Dunnett test (n = 3). Results are representative of two independent experiments. (b) B cells were stimulated with CpG-ODN2006 for 6 days, and proliferation of CD19^+^7AAD^-^ cells was assessed by flow cytometry. The graph on the right indicates the percentage of CFSE^low^ cells among CD19^+^7AAD^-^ cells. Among the CpG-ODN2006 stimulated samples, TGF-β treated cells were compared with TGF-β untreated cells using one way ANOVA, Dunnett test (n = 3). Results are representative of two independent experiments. (c) B cells were cultured under IL-21, sCD40L, CpG-ODN2006, and BCR stimulation for 4 days, and proliferation of CD19^+^7AAD^-^ cells was assessed by flow cytometry. The graph on the right indicates the percentage of CFSE^low^ cells among CD19^+^7AAD^-^ cells. Among the stimulated samples, TGF-β treated cells were compared with TGF-β untreated cells using one way ANOVA, Dunnett test (n = 3). Results are representative of two similar experiments. stim: IL-21, sCD40L, CpG-ODN2006, and BCR stimulation. (d) B cells were cultured under CpG-ODN2006 and BCR stimulation for 6 days, and the percentage of CD38^high^ plasmablasts among CD19^+^7AAD^-^ B cells was assessed by flow cytometry. TGF-β treated cells were compared with TGF-β untreated cells using one way ANOVA, Dunnett test (n = 3). Results are representative of two similar experiments.

In addition to interleukins and CD40L, Toll-like receptor (TLR) stimulation and B cell receptor (BCR) stimulation also play important roles in activating B cells; therefore, the effect of TGF-β1 and β3 was assessed in B cells cultured under TLR9 stimulation and BCR stimulation. Antibody production and B cell proliferation were inhibited by TGF-β1 and β3 in B cells cultured under TLR9 stimulation (Fig 3A and 3B), and TGF-β1 and β3 inhibited B cell proliferation and differentiation into CD38^high^ plasmablasts when B cells were stimulated with BCR agonists along with other stimulatory signals (Fig 3C and 3D).

### TGF-β3 Induces Phosphorylation of Smad1/5

Next, we sought to determine signal transduction pathways involved in B cell suppression by TGF-β3. The canonical pathway for signal transduction of the TGF-β superfamily is the Smad pathway. In general, TGF-β signals through Smad2 and Smad3, and Smad1 and Smad5 are involved in the transduction pathway of other members of the TGF-β superfamily, such as bone morphogenetic proteins (BMPs)[1]. However, it has been suggested that phosphorylation of Smad1 and Smad5 may be important for the suppression of human B cells by TGF-β1, and it has also been reported that Smad1/5 are phosphorylated upon TGF-β1 stimulation in human primary B cells[18].

**Fig 4:**
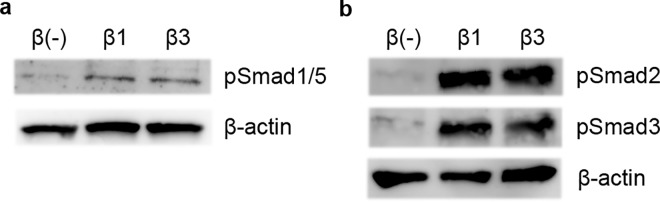
TGF-β3 induces phosphorylation of Smad 1 and Smad5. B cells were cultured overnight in serum free medium and stimulated with TGF-β1 or β3 for an hour. Phosphorylation of Smad1/5 (a) or Smad2 and Smad3 (b) were assessed by Western blotting. Results are representative of two similar experiments.

Therefore, we examined whether TGF-β3 induces phosphorylation of Smad1 and Smad5 as well. B cells were cultured overnight in serum free medium to exclude the effect of TGF-β in the serum and stimulated for an hour with TGF-β1 or β3. Like TGF-β1, TGF-β3 induced phosphorylation of Smad1 and Smad5 in primary human B cells (Fig 4A) in addition to Smad2 and Smad3 (Fig 4B).

### TGF-β1 and β3 Inhibit Various Molecules Important for B Cell Function

**Fig 5:**
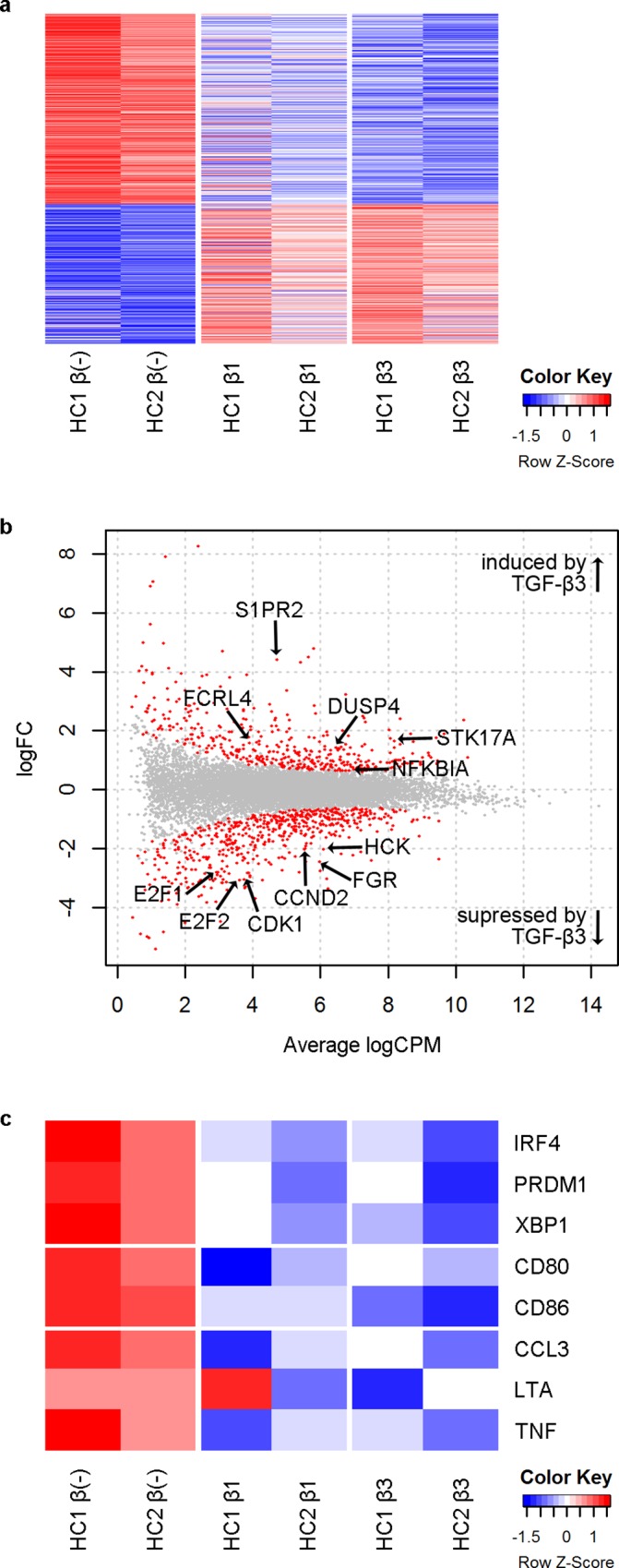
TGF-β1 and β3 inhibit various molecules important for B cell function. B cells from two healthy individuals (HC1 and HC2) were cultured for 48 hours under IL-21 and CD40L stimulation with or without TGF-β and transcriptome analysis was performed by RNA-Seq. (a) Heatmap of genes differentially expressed between TGF-β(-) samples vs. TGF-β3 samples. (b) MA plot comparing TGF-β(-) samples vs. TGF-β3 samples. Differentially expressed genes are shown in red, and names of selected genes are indicated. (c) Heatmap of genes important in B cell function.

To further elucidate the mechanism for B cell suppression by TGF-β1 and β3, B cells from healthy individuals were cultured under IL-21 and CD40L stimulation with or without TGF-β, and transcriptome analysis was performed by RNA-Seq. Both TGF-β1 and β3 induced profound changes in the transcriptome, and the changes induced by TGF-β1 and β3 were similar with the majority of genes differentially expressed by TGF-β3 having a tendency to be modulated in the same direction by TGF-β1 (Fig 5A).

Consistent with the inhibition of B cell proliferation by TGF-β3, TGF-β3 downregulated key molecules involved in the cell cycle such as E2F proteins, cyclin dependent kinase 1 (*CDK1*), and cyclin D2 (*CCND2*) (Fig 5B), and pathway analysis using IPA suggested that “proliferation of cells” and related pathways were suppressed by TGF-β3 (Table 1). TGF-β3 downregulated Src family kinases that are important in B cell proliferation, including Hck and Fgr[34]. *NFKBIA*, which encodes IκBα and inhibits NF-κB signaling, was induced by TGF-β3. Other negative regulators of B cell activation and survival, including *S1PR2*[35], *DUSP4*[36], *FCRL4*[37], and *STK17A*[38, 39], were also induced by TGF-β3 (Fig 5B).

**Table 1:** Diseases and Functions Predicted to be Inhibited by TGF-β3.

Diseases or Functions Annotation	p-Value	Activation z-score
cell proliferation of tumor cell lines	1.12x10^-21^	-4.04
proliferation of cells	8.90x10^-43^	-3.66
cell proliferation of breast cancer cell lines	5.79x10^-11^	-3.29
metabolism of DNA	1.35x10^-9^	-3.07
M phase of tumor cell lines	1.46x10^-10^	-2.92

IPA was used to predict diseases or functions inhibited by TGF-β3. p-Values indicated the degree of overlap between differentially expressed genes between TGF-β untreated and TGF-β3 treated samples and genes in the gene set by Fisher’s exact test, and z-scores are indicators of the activation or inhibition based on the Downstream Effects Analysis algorithm[31]. Five functions with the lowest activation z-scores are shown.

Further examination of genes important in B cell function showed that transcription factors (TFs) important in B cell differentiation into ASCs, including interferon regulatory factor 4 (*IRF4*), *PRDM1*, and X-box binding protein 1 (*XBP1*), were downregulated by both TGF-β1 and β3 (Fig 5C). In addition, TGF-β1 and β3 tended to suppress the expression of certain co-stimulatory molecules, pro-inflammatory cytokines, and chemokines, suggesting that TGF-β1 and β3 may inhibit B cell function as APCs and producers of pro-inflammatory cytokines and chemokines in addition to their role as ASCs (Fig 5C). Next, Ingenuity Pathway Analysis (IPA) was used to predict “upstream regulators” that could explain changes in the transcriptome induced by TGF-β3, and molecules important in B cell proliferation and differentiation, including FOXM1[40] and cRel[41], were predicted to be suppressed by TGF-β3 (Table 2).

**Table 2:** Molecules Predicted to be Suppressed by TGF-β3.

TBX2	FOXM1	CCND1	E2F3	MYC
MITF	SREBF1	SREBF2	FOXO1	E2F1
E2F2	ATF4	SOX2	TFDP1	ARNTL
IRF5	IRF3	REL	TP63	MED1
MBD2	SATB1	USF1	HMGB1	IRF1
MYBL2	NKX2-3	PPARGC1B	KLF15	MKL2
MAX	SIRT2	TLX1	HOXB4	

Upstream Regulator Analysis in IPA was used to predict molecules inhibited by TGF-β3[31]. Transcription regulators with activation z-scores less than 2 (i.e. whose function was predicted to be inhibited by TGF-β3) are shown.

### TGF-β1 and β3 Inhibit TFs Essential for B Cell Differentiation into ASCs

**Fig 6:**
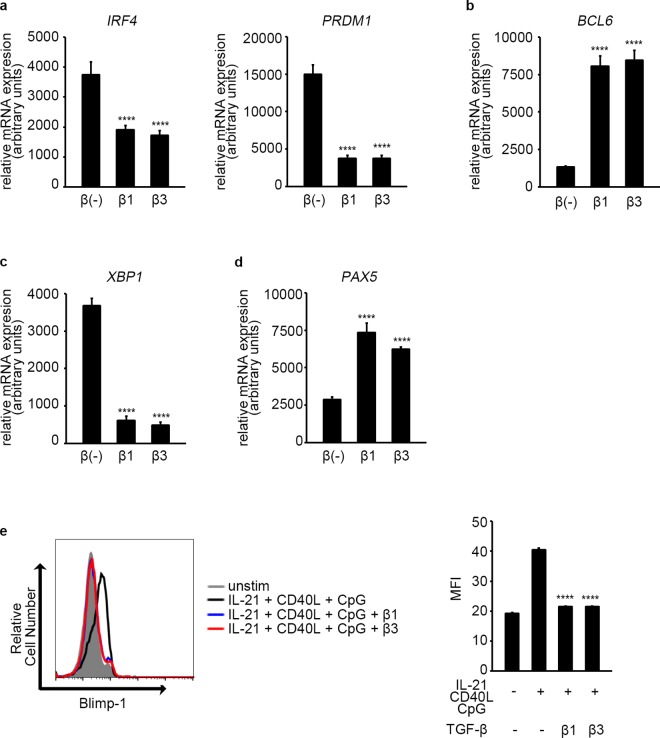
TGF-β1 and β3 inhibit TFs essential for B cell differentiation into ASCs. (a-d) B cells were cultured under IL-21 and CD40L stimulation for 3 days (a, b) or 5 days (c, d), and the expression of the indicated genes was assessed by quantitative PCR. Expression relative to *GAPDH* is shown. TGF-β treated cells were compared with TGF-β untreated cells using one way ANOVA, Dunnett test (n = 5). Results are representative of two (b), three (d), or four (a,c) independent experiments. (e) B cells were cultured under IL-21, CD40L, and CpG-ODN2006 stimulation for 4 days with or without TGF-β. Expression of Blimp-1 on CD19^+^ B cells was assessed by intracellular staining. TGF-β treated cells were compared with TGF-β untreated cells using one way ANOVA, Dunnett test (n = 3). Results are representative of three similar experiments.

In B cells, high levels of IRF4 induce the expression of B lymphocyte-induced maturation protein-1 (Blimp-1), and Blimp-1, in turn, induces various TFs essential for ASCs, such as XBP1[42]. As TGF-β1 and β3 suppress B cell differentiation into ASCs (Figs 1C and 3D) and RNA-Seq analysis suggested that TGF-β3 suppresses the expression of those TFs (Fig 5C), the effect of TGF-β1 and β3 on those TFs was examine further. Quantitative PCR confirmed that both TGF-β1 and β3 inhibit the expression of *IRF4*, *PRDM1*, and *XBP1*, and B cells treated with TGF-β1 or β3 failed to downregulate *BCL6* and *PAX5*, TFs that suppress differentiation into ASCs (Fig 6A–6D). Intracellular staining indicated that both TGF-β1 and β3 inhibit the expression of Blimp-1 at the protein level as well (Fig 6E).

### TGF-β1 and β3 Inhibit Phosphorylation of Syk

**Fig 7:**
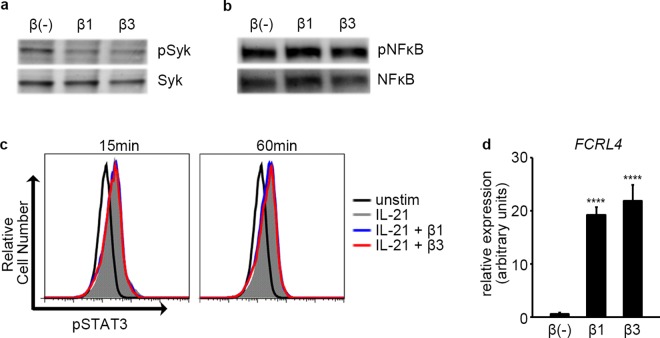
TGF-β1 and β3 inhibit phosphorylation of Syk. (a) B cells were cultured overnight without TGF-β or with TGF-β, and BCR stimulation was induced for 3 minutes. Results are representative of three similar experiments. (b) B cells were treated overnight with medium, TGF-β1, or TGF-β3 and stimulated with IL-21 and sCD40L for 3 minutes. Results are representative of two similar experiments. (c) B cells were treated overnight with medium, TGF-β1, or TGF-β3 and stimulated with IL-21 for the indicated times. Results are representative of two similar experiments. (d) B cells were cultured overnight without TGF-β or with TGF-β, and the expression of *FCRL4* was examined by quantitative PCR. Expression relative to *GAPDH* is shown. TGF-β treated cells were compared with TGF-β untreated cells using one way ANOVA, Dunnett test (n = 3). Results are representative of two similar experiments.

TGF-β3 has been reported to suppress phosphorylation of Syk, RelA, and STAT proteins in mice[25]. To further elucidate the mechanism for the inhibition of B cell function by TGF-β1 and β3 in humans, the effect of TGF-β1 and β3 on phosphorylation of Syk, RelA, and STAT3 was examined. B cells treated overnight with TGF-β1 and β3 showed decreased phosphorylation of Syk upon B cell receptor (BCR) stimulation (Fig 7A). However, phosphorylation of RelA was not inhibited by TGF-β1 or TGF-β3 in humans (Fig 7B). TGF-β1 and β3 did not substantially affect phosphorylation of STAT3 upon IL-21 stimulation either (Fig 7C).

To examine the mechanism for the inhibition of Syk phosphorylation by TGF-β1 and β3, the expression of the λ light chain and the κ light chain on the cell surface was examined on B cells cultured with TGF-β1 and β3. The expression of the λ light chain and the κ light chain did not change to a significant extent upon overnight culture with TGF-β1 or β3 (S1 Fig), suggesting that the decrease in phosphorylation of Syk was not due to reduced expression of the BCR complex on the cell surface. Fc receptor like 4 (FcRL4) has been reported to inhibit phosphorylation of Syk upon BCR stimulation by interacting with phosphatases, SHP-1 and SHP-2[37]. As FcRL4 has been implicated in TGF-β1 mediated suppression of B cell function in HIV infection[43] and RNA-Seq analysis suggested that TGF-β3 also induces the expression of FcRL4 (Fig 5B), we hypothesized that FcRL4 might also be involved in the inhibition of Syk phosphorylation by TGF-β3. Quantitative PCR confirmed that B cells cultured overnight with TGF-β1 and β3 upregulated *FCRL4*, suggesting that TGF-β1 and β3 may inhibit phosphorylation of Syk by upregulating FcRL4 (Fig 7D).

## Discussion

TGF-β3 is a cytokine whose importance has recently been recognized in immunology[23–25, 44] and inhibits the function of murine B cells[25]. The effect of TGF-β3 on human B cells has not yet been reported, and we herein examined the effect of TGF-β3 on human B cells. Notably, TGF-β3 inhibited B cell function as potently as TGF-β1. TGF-β3, like TGF-β1, induced phosphorylation of Smad1/5 in addition to Smad2 and Smad3. Transcriptome analysis and subsequent studies revealed that TGF-β1 and β3 suppress key molecules in B cell function such as IRF4, Blimp-1, XBP1, and Syk.

Interestingly, Smad1/5, which are usually known as mediators of BMPs signaling, not TGF-β signaling[1], was phosphorylated upon TGF-β1 and β3 stimulation in human primary B cells in addition to Smad2 and Smad3. Studies of B cell-specific Smad2 deficient mice[45] and Smad3 deficient mice[46] have suggested that Smad2 and Smad3 are not necessary for the inhibition of B cell proliferation by TGF-β1. Among human B cell lymphoma cell lines, TGF-β1 mediated suppression of cell proliferation is seen only in cell lines in which Smad 1/5 are phosphorylated upon TGF-β1 treatment[18]. These studies suggest that Smad1/5 might be important for TGF-β1 mediated suppression of B cells, and our data that TGF-β3 induces Smad1/5 phosphorylation is in line with these reports and also suggests the possibility that Smad1/5 might be involved in the inhibition of B cell function by TGF-β1 and β3.

In murine B cells, it has been reported that Id3, which is induced by Smad1/5, induces growth arrest and apoptosis by antagonizing E proteins[47, 48], and the induction of Id3 by Smad1/5 may also be responsible for the inhibition of human B cells by TGF-β1 and β3. Although it has been reported that Smad signaling and signals further downstream differs between TGF-β1 and β3 in murine T cells[23], in human B cells, phosphorylation of Smad and changes in the transcriptome induced by TGF-β1 and β3 were similar.

It has been reported that the proliferation of B cells is essential for B cell differentiation into ASCs[49, 50], and both TGF-β1 and β3 strongly inhibited B cell proliferation and subsequently differentiation into CD38^high^ plasmablasts, as well as the expression of key TFs in the differentiation of ASCs, including IRF4, Blimp-1, and XBP1. It has been reported that NFκB plays an important role in the induction of IRF4 in B cells[51] and as RNA-Seq analysis suggested that TGF-β3 induces the expression of IκBα, the suppression of NFκB signaling by IκBα may play a role in the inhibition of B cell differentiation into ASCs by TGF-β1 and β3.

In addition to IRF4, Blimp-1, and XBP1, phosphorylation of Syk was inhibited by TGF-β1 and β3. FcRL4, which inhibits BCR signaling[37] and has been suggested to mediate B cell inhibition by TGF-β1[43], was also upregulated by TGF-β3; therefore, FcRL4 might be involved in the inhibition of BCR signaling by TGF-β3 as well. Further studies are necessary to determine the exact mechanism for the suppression of BCR signaling by TGF-β1 and β3 and its relationship to Smad signaling.

In general, the effect of TGF-β1 and β3 on human B cells was similar to those reported for murine B cells; however, there were significant differences. For example, in murine B cells, TGF-β3 has been reported to inhibit phosphorylation of RelA[25], but in human B cells, TGF-β1 and β3 did not inhibit phosphorylation of RelA. There may be other differences in the mechanism of B cell suppression by TGF-β1 and β3 between murine and human B cells, and the mechanism for the suppression of human B cells by TGF-β1 and β3 needs to be investigated further. In addition, it has been reported that TGF-β1 enhances B cell proliferation and IgA secretion under certain circumstances [15], and the effect of TGF-β3 on human B cells under those conditions needs further investigation.

In summary, TGF-β3 suppresses human B cell function as potently as TGF-β1 by suppressing key molecules in B cell function including Syk, IRF4, Blimp-1, and XBP1. TGF-β3 has been suggested to be therapeutic in a mouse model of SLE[25], and the findings of this study suggest that TGF-β3 modifying therapy may be therapeutic in human autoimmune diseases with B cell dysregulation. It has been suggested that B cells with increased Syk phosphorylation might be a source for pathogenic plasma cells in SLE[52], and TGF-β3, which inhibits phosphorylation of Syk and ASC differentiation, may be able to inhibit those B cells as well as their differentiation into pathogenic plasma cells.

The effects of TGF-β1 and β3 on other cell types are different, and TGF-β3 modifying therapy may be more suitable for treatment of autoimmune diseases compared to TGF-β1 for several reasons. First, it has been suggested that TGF-β3 has a better effect on glucose tolerance compared to TGF-β1[53]. In addition, TGF-β1 is associated with cutaneous wound healing with scaring while TGF-β3 is associated with wound healing without scarring[6, 8], and TGF-β1 promotes fibrosis in the lung while TGF-β3 does not[7]. Therefore, an augmentation of TGF-β3 activity could be a potential therapeutic strategy for autoimmune diseases while avoiding glucose intolerance and scarring and fibrosis of other tissues, which might be induced by TGF-β1 modifying therapy. Thus, our study suggests TGF-β3 as a potential new target for therapy in autoimmune diseases.

## Supporting Information

S1 Fig TGF-β1 and β3 do not alter the expression of light chains on the cell surface. B cells were cultured overnight with or without TGF-β, and the expression of the λ light chain and the κ light chain was examined by flowcytometry. Results are representative of two similar experiments.
(TIF)
