# Supplementary figures and images for: TGF-β3 Inhibits Antibody Production by Human B Cells
Source: PLoS One. 2017 Jan 4;12(1):e0169646. doi: 10.1371/journal.pone.0169646 (PMC5215424; doi:10.1371/journal.pone.0169646)

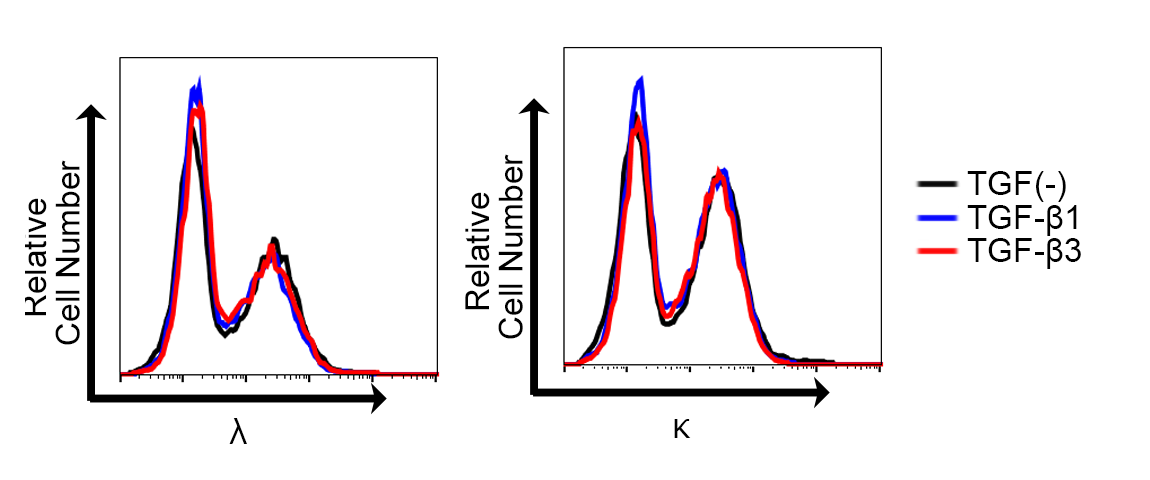

Supplement: S1 Fig — B cells were cultured overnight with or without TGF-β, and the expression of the λ light chain and the κ light chain was examined by flowcytometry. Results are representative of two similar experiments. (TIF) [file pone.0169646.s001.tif]
